# Nanoemulsions as Delivery Systems for Poly-Chemotherapy Aiming at Melanoma Treatment

**DOI:** 10.3390/cancers12051198

**Published:** 2020-05-09

**Authors:** Chiara Dianzani, Chiara Monge, Gianluca Miglio, Loredana Serpe, Katia Martina, Luigi Cangemi, Chiara Ferraris, Silvia Mioletti, Sara Osella, Casimiro Luca Gigliotti, Elena Boggio, Nausicaa Clemente, Umberto Dianzani, Luigi Battaglia

**Affiliations:** 1 Department of Drug Science and Technology, University of Turin, via Pietro Giuria 9, 10125 Turin, Italy; chiara.dianzani@unito.it (C.D.); chiara.monge123@gmail.com (C.M.); gianluca.miglio@unito.it (G.M.); loredana.serpe@unito.it (L.S.); katia.martina@unito.it (K.M.); luigi.cangemi@unito.it (L.C.); chiara.viola.isabella@gmail.com (C.F.); 2 Department of Veterinary Sciences, University of Turin, Largo Paolo Braccini 2, 10095 Grugliasco, Italy; silvia.mioletti@unito.it; 3 San Giovanni Bosco Hospital, Piazza del Donatore di Sangue 3, 10154 Turin, Italy; sara.osella@aslcittaditorino.it; 4 Department of Health Sciences and Interdisciplinary Research Center of Autoimmune Diseases (IRCAD), University of Eastern Piedmont (UPO), via Solaroli 17, 28100 Novara, Italy; luca.gigliotti@med.uniupo.it (C.L.G.); elena.boggio@med.uniupo.it (E.B.); nausicaa.clemente@med.uniupo.it (N.C.); umberto.dianzani@med.uniupo.it (U.D.)

**Keywords:** nanoemulsions, Intralipid^®^, temozolomide, rapamycin, bevacizumab, melanoma

## Abstract

*Aims:* Advanced melanoma is characterized by poor outcome. Despite the number of treatments having been increased over the last decade, current pharmacological strategies are only partially effective. Therefore, the improvement of the current systemic therapy is worthy of investigation. *Methods:* a nanotechnology-based poly-chemotherapy was tested at preclinical level. Temozolomide, rapamycin, and bevacizumab were co-loaded as injectable nanoemulsions for total parenteral nutrition (Intralipid^®^), due to suitable devices, and preliminarily tested in vitro on human and mouse cell models and in vivo on the B16-F10 melanoma mouse model. *Results:* Drug combination was efficiently loaded in the liquid lipid matrix of Intralipid^®^, including bevacizumab monoclonal antibody, leading to a fast internalization in tumour cells. An increased cytotoxicity towards melanoma cells, as well as an improved inhibition of tumour relapse, migration, and angiogenesis were demonstrated in cell models for the Intralipid^®^-loaded drug combinations. In preliminary in vivo studies, the proposed approach was able to reduce tumour growth significantly, compared to controls. A relevant efficacy towards tumour angiogenesis and mitotic index was determined and immune response was involved. *Conclusions:* In these preliminary studies, Intralipid^®^ proved to be a safe and versatile poly-chemotherapy delivery system for advanced melanoma treatment, by acting on multiple mechanisms.

## 1. Introduction

If timely diagnosed, early stage melanoma can be treated by surgery, allowing an almost complete recovery. In contrast, if neglected or unidentified, it can progress up to the IV stage, which is the most advanced and lethal disease stage [1], characterized by metastases and relapses, even after a previous surgical removal. By spreading beyond the regional lymph-nodes, it can reach sites distal to the primary lesion, including lungs, brain, and bones [2]. Current treatments, based on novel pharmacological strategies, have significantly improved clinical outcomes. Nowadays, apart from classical dacarbazine treatment, which can be considered only as a palliative care, several targeted therapies can be offered to patients with melanoma, depending on their mutational status and disease burden [3]. Treatment with BRAF inhibitors (BRAFi) is associated with high response rates [4]. However, the median time-to-progression remains limited to 5.1–8.8 months [5]. The addition of a MEK inhibitor (MEKi) is expected to improve slightly this clinical endpoint [6], but does not overcome chemoresistance, which remains a major clinical problem. Within this concern, several mechanisms have been proposed, including activation of other molecular pathways associated with cell growth, such as the PI3K/PTEN/Akt/mTOR pathway [7,8]. Furthermore, therapeutic monoclonal antibodies directed toward the T-lymphocyte-associated protein 4 (CTLA-4) or the programmed cell death protein 1 (PD-1) receptors are now approved in many countries as valuable treatments to unlock the anti-tumour immune response. However, they appear to be effective only in patients with pre-existing anti-tumour immunity, whereas in patients without such pre-existing immunity, these drugs fail to promote a beneficial anti-tumour immune response de novo [9]. Thus, after failing targeted and immune therapies (which still occurs in the majority of patients), limited treatment options remain. Therefore, the improvement of the pharmacological strategies currently available is worthy of investigation.

Nanotechnology allows the potential improvement of the effectiveness of chemotherapeutics, overcoming their drawbacks and side effects [10,11]. Injectable lipid nanoemulsions for total parenteral nutrition (Intralipid^®^-IL) have been clinically used as a source of essential fatty acids and vitamins. Recent interest in utilizing lipid nanoemulsions to deliver lipid soluble therapeutic agents intravenously has been continuously growing, due to the biocompatible nature of the lipid-based delivery systems, as well as to their nanometric size range, that influences their biological properties [12].

Noteworthy, thanks to a combination of agents acting via different mechanisms of actions, poly-chemotherapy could allow the dose of the single drugs to be lowered, thus reducing exposure, side effects, and significantly increasing clinical response. The choice of the drugs to be co-administered is driven by mechanistic considerations.

This work investigated a new nanotechnology-based poly-chemotherapy, that employs temozolomide (TMZ), rapamycin (RAP), and bevacizumab (BVZ) co-loaded in injectable nanoemulsions, for the treatment of advanced stage melanoma, through in vitro and in vivo studies on available cell and animal models. TMZ is an alkylating agent which is currently a second line agent for melanoma. In contrast to dacarbazine, it can cross the blood-brain barrier, thus targeting even brain metastasis. The effects of TMZ against melanoma can be improved by loading it in solid lipid nanoparticles, through formation of a dodecyl ester (TMZ-C12) [13]. The effects of TMZ could be even improved when combined with mTOR inhibitors, as shown by a single-arm Phase II study evaluating the effects of TMZ plus the rapamycin (RAP) analogue everolimus [14], which shows that everolimus decreased chemoresistance to TMZ, likely in an mTOR-dependent manner [15]. BVZ is a humanized monoclonal antibody with anti-angiogenetic activity, acting by binding to vascular growth factor (VEGF). The VEGF-mediated angiogenesis can be decreased by RAP, since it inhibits soluble CD40L-mediated transactivation of VEGF; this mechanism seems to be clinically relevant regarding the tumour growth [16]. Moreover, the combination of BVZ and RAP derivative everolimus was found to have moderate activity and was well tolerated in patients with metastatic melanoma [17].

## 2. Results

### 2.1. Biopharmaceutical Characterization of IL MIX

Physico-chemical properties of the combination of drugs co-loaded in IL (IL MIX) were initially evaluated. As shown in Table 1, a negatively charged nanoemulsion was obtained, with good recoveries and percent drug entrapment efficiency (EE%) for each of the three drugs loaded.

**Figure 1:**
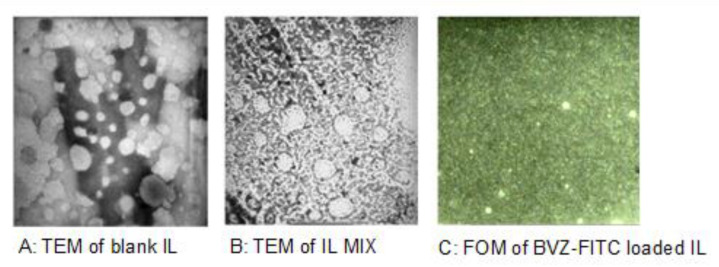
Microscopical characterization of IL (TEM 105,000× magnification), IL MIX (TEM 145,000× magnification) and IL BVZ-FITC (FOM 630× magnification). (**A**) TEM of blank IL; (**B**) TEM of IL MIX; (**C**) FOM of BVZ-FITC loaded IL. IL: Intralipid^®^; IL MIX: combination of drugs co-loaded in IL; BVZ-FITC: fluorescein isotiocyanate labelled bevacizumab; TEM: transmission electronic microscopy; FOM: fluorescence optical microscopy.

BVZ entrapment in the IL oil droplets, obtained through an ion pairing approach [18], was further confirmed by employing BVZ labelled with fluorescein isotiocyanate (FITC; BVZ-FITC; Figures S4.1 and S5, Tables S3 and S4). In particular, in the field flow fractionation (FFF) fractogram of IL BVZ-FITC, the fluorescence signal from BVZ-FITC meets the UV-vis/light scattering signal from IL nanodroplets, thus confirming the effective loading of the antibody in IL (Figure S4.2). Furthermore, with fluorescence optical microscopy (FOM) the dotted fluorescence pattern of BVZ-FITC overlapped the oil droplets. Consistently, when IL MIX was examined with transmission electron microscopy (TEM), BVZ was detectable as condensed material at the surface of the oil droplets (Figure 1).

In order to investigate the stability of the nanoemulsion at different pH conditions, to those in the bloodstream and those in the acid tumour microenvironment, the same was diluted in 2 mM phosphate buffer at various pH values, and kept under stirring for 24 h: then particle size and Zeta potential were measured. Particle size was maintained without droplet aggregation, while Zeta potential became more negative by increasing the pH value, owing to deprotonation of the phosphate group of the lecithin emulsifier (Supplementary Materials Section S1-Table S1).

Cell (A2058 and B16-F10) internalization of fluorescently labelled IL and drugs (loaded in IL) was quantified through flow cytometry (Table 2). Despite the different fluorescence intensities of the formulations under study, in comparison to untreated cells, significantly higher integrated mean fluorescence intensity (iMFI) values were measured in both the cell lines, indicating a fast internalization of lipid droplets and loaded drugs. 6-Coumarin was chosen as fluorescent probe for the IL lipid matrix [19,20]. Instead, internalization of RAP and BVZ loaded in IL was investigated, after fluorescent labelling, because of their different physico-chemical properties. TMZ was not directly investigated, because in cells it undergoes the aforementioned pH-dependent degradation [13], leading to a quantification not reproducible. 

### 2.2. Pharmacological Characterization of IL MIX: In Vitro Studies

#### 2.2.1. Inhibition of Cell Growth

**Figure 2:**
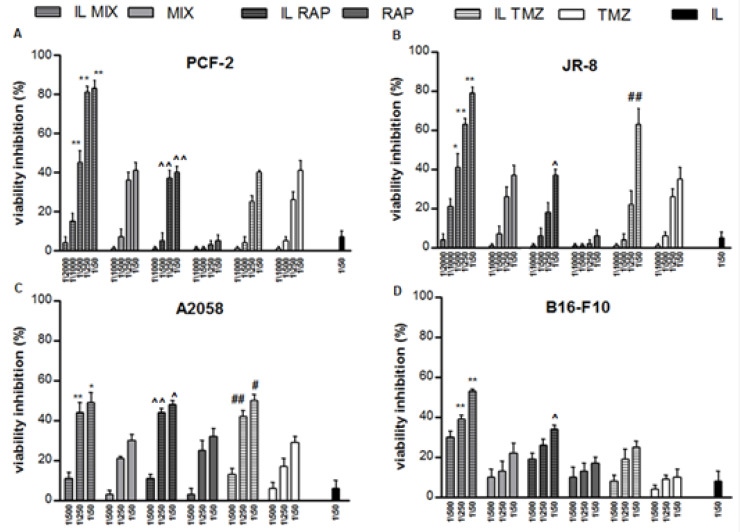
Cytotoxicity experiments towards melanoma cell lines; IL MIX vs. MIX * *p* < 0.05 ** *p* < 0.01; IL RAP vs. RAP ^ *p* < 0.05 ^^ *p* < 0.01; IL TMZ vs. TMZ # *p* < 0.05 ## *p* < 0.01. IL: Intralipid^®^; MIX: combination of drugs; IL MIX: MIX co-loaded in IL; RAP: rapamycin; IL RAP: RAP loaded in IL; TMZ: temozolomide; IL TMZ: TMZ dodecyl ester loaded IL.

In order to characterize the antitumoral activity of the IL MIX, its effects on a panel of well-characterized melanoma cell lines were studied. First, the cytotoxic effects (WST-1 assay) of IL MIX were measured and compared to those exerted by RAP loaded IL (IL RAP), TMZ-C12 loaded IL (IL TMZ), blank IL, free RAP, free TMZ and free MIX. Data from free BVZ and BVZ loaded IL (IL BVZ) are not shown, since no effect on cell viability was measured. As shown in Figure 2, both free TMZ and RAP exerted cytotoxic effects in a concentration-dependent manner. In addition, in comparison to the free drugs, significantly higher effects were measured when drugs were loaded in IL and even more pronounced when they were combined (IL MIX).

**Figure 3:**
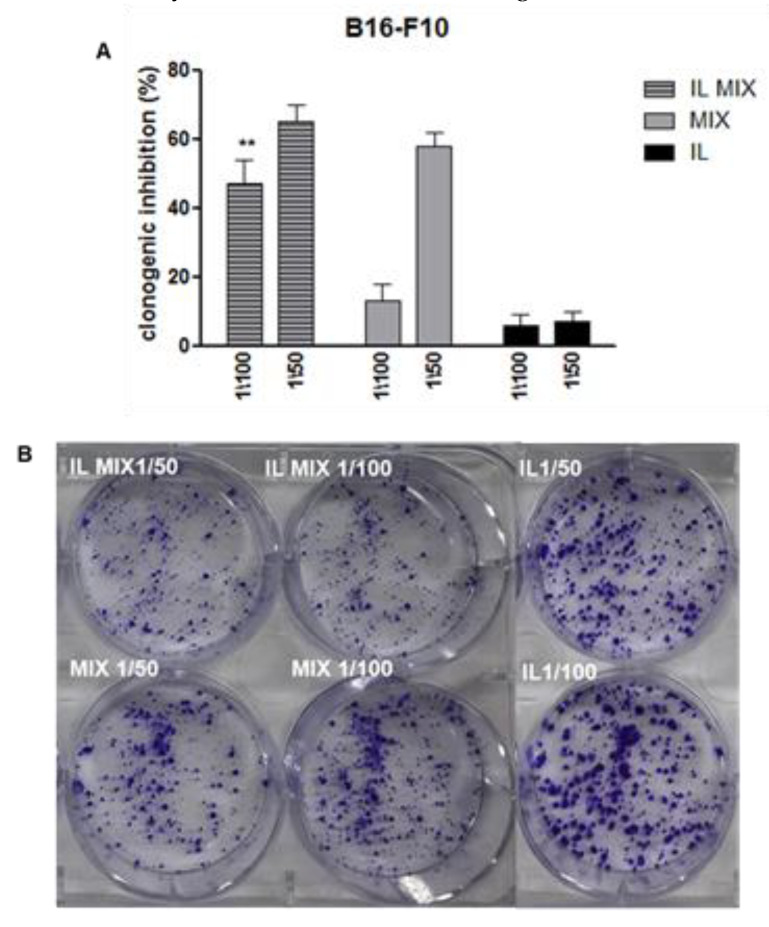
Clonogenic assay in B16-F10 cells. ** IL MIX vs. MIX *p* < 0.01; (**A**) quantification; (**B**) wells stained with crystal violet. IL: Intralipid^®^; MIX: combination of drugs; IL MIX: MIX co-loaded in IL

These effects were confirmed and extended when clonogenic assays were performed. As shown in Figure 3, exposure of B16-F10 cells to IL MIX exerted significantly higher effects than the combination of free agents (MIX). As indicated by these results, loading of TMZ, RAP, and BVZ into IL could increase their ability to contrast melanoma cell growth.

#### 2.2.2. Inhibition of Cell Migration and Angiogenesis

**Figure 4:**
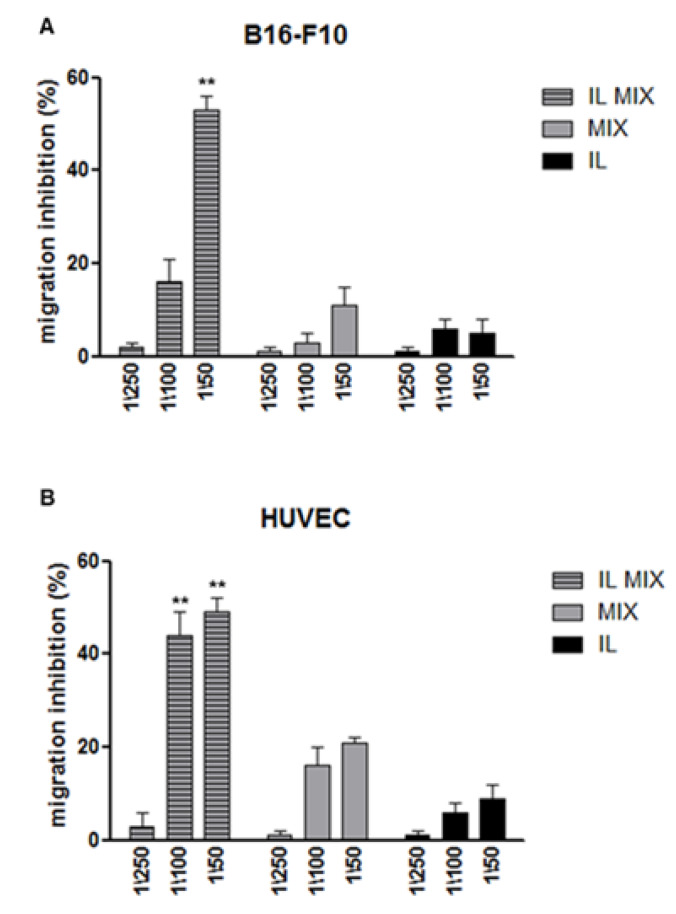
Migration assay. ** IL MIX vs. MIX *p* < 0.01; (**A**) B16-F10 cells; (**B**) HUVEC. IL: Intralipid^®^; MIX: combination of drugs; IL MIX: MIX co-loaded in IL.

**Figure 5:**
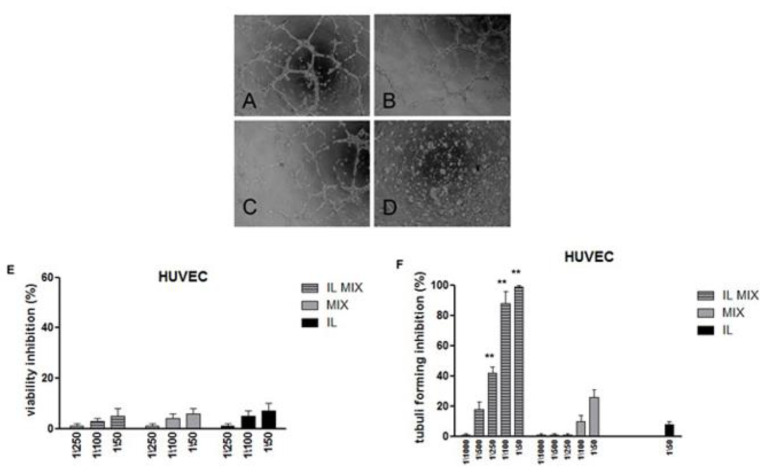
Tube formation assay; (**A**) Ctrl; (**B**) IL 1:50; (**C**) MIX 1:50; (**D**) IL MIX 1:50; (**E**) 6 h viability inhibition on HUVEC; (**F**) quantification of tube forming assay (6 h); ** IL MIX vs. MIX *p* < 0.01. IL: Intralipid^®^; MIX: combination of drugs; IL MIX: MIX co-loaded in IL.

In order to better ascertain the anti-tumoral activity of IL MIX, its effects on cell migration (Boyden chamber assay) and endothelial tube formation were investigated. For these experiments, B16-F10 cell line was employed as a representative tumoral cell model. As shown in Figure 4 (cell migration) and in Figure 5 (tube formation), in comparison to both blank IL and MIX, significantly higher effects were exerted by IL MIX, thus suggesting that a more pronounced anti-tumoral effect could be exerted by drugs when combined and loaded in IL. A preliminary viability experiment adopting a 6 h incubation period excluded that the cytotoxity of formulations under study could affect migration or the tube formation process.

### 2.3. Pharmacological Characterization of IL MIX: In Vivo Studies

**Figure 6:**
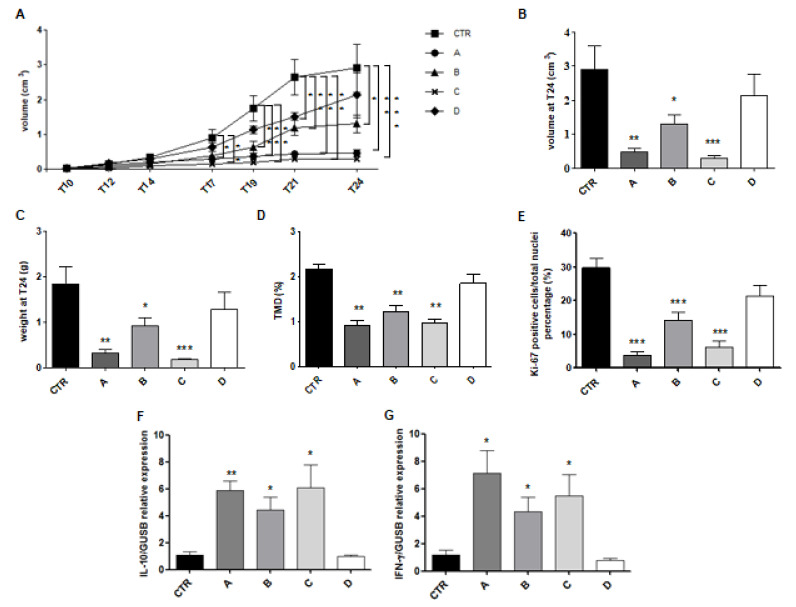
Results of animal experiments. Statistical analysis: (**A**) Tumour volume: formulations vs. CTR * *p* < 0.05; ** *p* < 0.01; *** *p* < 0.005; (**B**) Tumour volume at endpoint: CTR vs. B * *p* < 0.05; CTR vs. A ** *p* < 0.01; CTR vs. C *** *p* < 0.005; (**C**) Tumour mass at endpoint: CTR vs. B * *p* <0.05; CTR vs. A ** *p* < 0.01; CTR vs. C *** *p* < 0.005; (**D**) CD31 quantification: CTR vs. A, B, C ** *p* < 0.01; (**E**) Ki67 quantification: CTR vs. A, B, C *** *p* < 0.005; (**F**) IL-10 quantification: CTR vs. A ** *p* < 0.01; CTR vs. B, C * *p* < 0.05; (**G**) IFN-γ quantification: CTR vs. A, B, C * *p* < 0.05. CTR: control; A: Intralipid^®^ (IL) loaded with combination of drugs (MIX) and high dose of rapamycin (RAP); B: IL MIX-RAP low dose; C: MIX-RAP high dose; D: MIX-RAP low dose.

Results from in vitro experiments were instrumental in designing and performing a preliminary evaluation of IL MIX on B16-F10 mouse model of melanoma. In particular, the effects of two formulations of IL MIX, IL MIX-RAP high dose (formulation A) and IL MIX-RAP low dose (formulation B), were compared to the corresponding combinations of free drugs, MIX-RAP high dose RAP (formulation C) and MIX-RAP low dose (formulation D). In addition, phosphate buffered saline (PBS) was used as control (CTR). Results are summarized in Figure 6 and in the Supplementary Materials Section S2 (Figures S1.1 and S1.2). No animal was excluded due to excessive sufferance. Formulations A and C exerted significant effects on all the measured parameters, regardless of the presence of IL, probably because of the levelling effect of high dose RAP; differences between formulation B and D, instead, can be attributed to the drug delivery system employed.

## 3. Discussion

### 3.1. Technological Aspects

**Figure 7:**
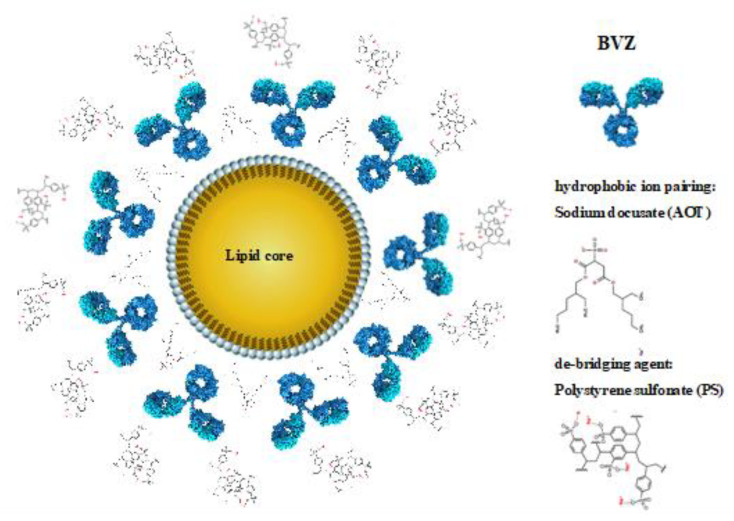
Scheme of the interaction between Intralipid^®^ (IL) and bevacizumab (BVZ).

Besides employment for total parenteral nutrition, IL has also been investigated for drug delivery purposes [12] but its usage as a vehicle for a poly-chemotherapy is innovative. In order to exploit the advantages of IL nanocarrier, lipophilic drugs should be loaded in the inner oily phase of the nanoemulsion. In the purposed poly-chemotherapy, only RAP is lipophilic. TMZ, instead, was “lipophilized” to an ester prodrug (TMZ-C12), which was demonstrated to increase its stability in the bloodstream, as reported in our previous research [13]. In fact, at neutral pH, free TMZ is rapidly hydrolysed to 5-(3-dimethyl-1-triazenyl) imidazole-4-carboxamide (MTIC), which is then quickly converted into its reactive form, the methyl-diazonium ion, that primarily methylates guanine residues of the DNA molecule. Lipophilic TMZ-C12 is almost insoluble in water and can be loaded within the oily core of IL (Table 1), which prevents the water-dependent imidazo–tetrazine ring cleavage. Furthermore, IL MIX was formulated by adding a small amount of pH = 3.0 citrate buffer, to avoid the potential degradation of TCMZ-C12, due to its slight partitioning in the outer phase. Therefore, despite a long-time stability assessment not being performed in this preliminary study, this strategy was effective in maintaining drug recovery above 90% after one month for TMZ-C12, as well as for other drugs co-loaded in IL. On the other hand, BVZ was loaded in the lipid phase of IL through an ion pairing strategy, previously developed by our research group, to load antibodies in SLN [18]. An ion pair forms thanks to the interaction between the positively charged amino groups of the antibody and the negatively charged groups of sodium docusate (AOT) surfactant, with a molar ratio 1:150 BVZ/AOT. However, while the lipid matrix of SLN is solid, in IL the inner lipid phase is liquid, causing AOT counter-ion to migrate at the surface of oil droplets. Thus, the multiple positive charges, displayed on high molecular weight BVZ, interact with the surrounding AOT coated droplets, leading to mutual aggregation. In order to avoid this phenomenon, polystyrene sulfonate (PS) was added as de-bridging agent, aiming to mask the excess positive charge at the droplet surface. Several pieces of evidence were provided to demonstrate the association between BVZ and IL oil droplets, including FFF (Figure S4.2), FOM (Figure 1C) and TEM (Figure 1B). In particular, in the presence of BVZ, condensed materials at the surface of oil droplets was noted in TEM images. This might confirm the aforementioned loading mechanism, which is summarized in Figure 7.

EE% of drugs in IL MIX was determined after size exclusion with Agarose CL 4B (Table 1). Despite frequently employed for SLN [13,18], this method is innovative for nanoemulsions. It is noteworthy that the measured EE% could be under-estimated because, in nanoemulsions, the lipid matrix is liquid whereas, in SLN, the drug is entrapped in the solid lipid core. Thus, we can hypothesize that a continuous drug partition occurs between the lipid phase and the mobile phase of IL during elution in the size exclusion column, with a partial drug leakage from the inner phase of IL.

### 3.2. Pharmacological Aspects

Efficient loading of the drugs in the liquid lipid matrix is not an easy task but loading in IL should lead to a fast internalization in tumour cells. In vivo, cellular uptake of IL after extravasation has been documented for endothelial cells and hepatocytes [21,22]. CD36/caveolin-mediated endocytosis is the main internalization mechanism [23] and might be effective also in tumour cells. According to the calculated iMFI values, IL-loaded drugs are rapidly internalized, regardless of the hydrophilic/lipophilic character and loading method, likely because drug internalization is mediated by loading in IL oil droplets.

Loading of the agents in IL was associated with the increased anti-tumoral effects measured by in vitro cytotoxicity, cell migration, and tube formation assays. In cell studies, dose ratios were optimized owing to literature data. Based on our previous experience [13], TMZ-C12 loaded in lipid vehicle exerts higher effects compared to the parent compound (TMZ), allowing a substantial reduction of the dose, ranging between 0.825 and 33 µM (0.3–12 µg/mL TMZ-C12 corresponding to 0.15–6 µg/mL pure TMZ). A sub-therapeutic BVZ dose range (3.3–132 nM; 0.5–20 µg/mL) was employed in order to point-out the effect of delivery in IL [18]. RAP was used at low concentrations (0.125–5 nM; 0.125–5 ng/mL) to avoid an excessive effect on cell viability [15,16]. Three human melanoma cell lines, with different phenotypes (including variable resistance to BRAFi), and a mouse cell line were used. Of note, response to free TMZ and RAP varied among human cell lines. These differences can be tentatively explained considering the inter-cell line variability (e.g., in terms of species, mutational status and/or activity at biochemical/molecular pathways). For example, being A2058 cells characterized by PTEN loss, they should be more sensitive to free RAP, compared to JR8 and PCF-2 [24]. All of the results obtained indicate the superiority of IL MIX compared to free MIX in blocking the oncogenic phenomena. In particular, in migration and tube formation assay BVZ was employed at sub-therapeutic concentrations [18]. Thus, even if employed in combination with TMZ and RAP, BVZ was inactive as free molecule (MIX), but effective when loaded in IL together with the other drugs (IL MIX).

In the in vivo setting (Figure 6), the TMZ-C12 dose was calculated similarly to our previous paper (1.6 mg/kg TMZ equivalent) [13], with a striking dose reduction compared to literature [25,26,27,28]. BVZ dose employing (5 mg/kg) was in line with the therapeutic range [29]. In order to investigate the possibility of using IL nanomedicine to modulate drug doses, based on the personal therapeutic needs, we employed RAP at two dose regimens. In formulation A, IL MIX was prepared under the same conditions used for in vitro studies, where a low dose of RAP was loaded [15,16]: In this case RAP dose administered to the animal model is 1.25 μg/kg. In formulation B, RAP loaded in IL MIX was increased by 1000 folds, in order to reach a 1.25 mg/kg dose in vivo [16,30,31]. Formulations C and D were the corresponding MIX with free drug combinations. High dose RAP formulations (A and C) exerted higher effects due to RAP on all the outcomes considered. However, this depends also on the tumour model and administration protocol, and different experimental settings could overcome this “levelling” effect by RAP. With low dose RAP formulations (B and D), differences between IL loaded and free poly-chemotherapy could be better appreciated. In particular, formulation B was associated with an important decrease in the percentage of TMD (CD31) compared to controls, indicating a decreased intra-tumour angiogenesis, in agreement with tube formation assay results. Secretion of IL-10 and IFN-γ was greatly increased after treatment with A, B, C formulations compared to controls. According to a generally accepted model, IL-10 is considered an anti-inflammatory and pro-oncogenic cytokine, associated to T regulator lymphocyte increase and M2 polarization of tumour associated macrophages. Conversely IFN-γ is produced by lymphocytes with anti-tumour and pro-inflammatory activity, such as type-1 T helper lymphocytes, cytotoxic T lymphocytes, and natural killer (NK) cells. Therefore, in the tumour microenvironment IL-10 and IFN-γ are generally seen as mutually antagonist cytokines [32]. However, IL-10 and IFN-γ secretion should be regulated by mechanisms other than a simple antagonism, since both are involved in the inflammation process, IFN-γ primarily acting as a triggering agent, while IL-10 in tissue repair. Furthermore, numerous studies show that, in an appropriate situation, IL-10 can promote IFN-γ production and even increase cytotoxic anti-tumour lymphocytes growth and tumour rejection [33]. Therefore, an adequate anti-tumour response requires a balanced production of both cytokines, and treatment with A, B, and C formulations is probably capable of favouring a resumption of the anti-tumour immune response.

### 3.3. Innovation and Future Perspectives

Currently, nanoemulsions employed in drug delivery can be classified as: phospholipid-based, PEGylated and cationic [34]. The first category includes nanoemulsions for total parenteral nutrition as such, which has been used to deliver several drugs, in both commercial (Diprivan^®^, Etomidat-Lipuro^®^, Limethason^®^, Restasis^®^, Fluad^®^) and experimental settings (paclitaxel, docetaxel, chlorambucil, curcumin, cinnarizine, bupivacaine, palmitoylrhixozin) [35,36,37,38,39,40,41]. The main field of application of “therapeutic” IL concerns anaesthetics [42], but interest is growing on anti-tumoral drugs, where improved pharmacokinetics, increased efficacy and reduced side effects are considered potential goals. A key innovation in our approach is the use of IL for the delivery of a combination of drugs, including high molecular weight antibodies, loaded in IL through HIP. Poly-chemotherapy loaded in IL can allow dose reduction of each drug, improving therapeutic effects and minimizing side effects. The selected drug combination loaded in IL exerted encouraging effects in both cellular and animal models, likely modulating different processes involved in tumour proliferation, spreading and angiogenesis. Future studies would investigate the influence of varying the chemical composition of the nanoemulsion (long chain vs. medium chain triglycerides) [43] and the therapeutic doses. Furthermore, since the immune system seems to play an important role in our in vivo setting, alternative melanoma models should be investigated, including xenografts, where the role of the immune response is minimized, to assess the role of reactivation of the anti-tumour immune response.

## 4. Materials and Methods

### 4.1. Nanoemulsions 

#### 4.1.1. Formulation

TMZ (Sigma-Aldrich, St. Louis, MO, USA) was lipophilized through formation of a dodecyl ester (TMZ-C12) [13,44,45] (see Supplementary Materials Section S3-Figure S2). Loading in IL 10% (Fresenius Kabi, Bad Homberg, Germany). IL of the combination of drugs (IL MIX) was obtained as follows: 2 mL IL was added to 40 μL buffer citrate 0.1 M pH = 3.0, in order to stabilize TMZ-C12, which was dissolved in dimethylformamide (DMF; 1.2 mg in 100 μL) and added dropwise (0.6 mg/mL, 1.65 mM TMZ-C12). A stock nanoemulsion of RAP was prepared by dissolving 1 mg RAP (Alfa-Aesar, Haverhill, MA, USA) in 4 mL IL, then 2 μL of stock nanoemulsion was added to the final IL formulation (0.25 μg/mL, 0.25 μM RAP). AOT stock solution (100 μL, 4.5 mg/mL) (Merck, Darmstadt, Germany), and then 80 μL of Avastin^®^ (Roche-Genentech, South San Francisco, CA, USA) were added to the final IL formulation (1 mg/mL, 6.6 μM BVZ), in order to form an ion pair at a 1:150 AOT–BVZ molar ratio [18]. Droplet aggregation was inhibited by adding 100 μL of 10 mg/mL solution of PS (Sigma-Aldrich, St. Louis, MO, USA) in water, as de-bridging agent.

Controls for cell studies were prepared as follows: IL TMZ-C12, IL RAP, IL BVZ were formulated to evaluate the effect of single drugs delivered in IL. Free drug solutions were prepared as follows: free TMZ and RAP were dissolved in dimethylsulphoxide (DMSO; 10 mg/mL—52 mM and 9.1 mg/mL—10 mM respectively), and Avastin^®^ was used as is (25 mg/mL—160 μM). The mixture of free drugs (MIX) was prepared by mixing the single stock solutions in suitable ratios.

For animal experiments an RAP high dose IL MIX formulation was also prepared, by employing 0.25 mg/mL (0.25 mM) RAP in the final IL formulation. MIX controls (low and high dose RAP) were prepared by dissolving RAP (either 0.25 μg/mL or 0.25 mg/mL, respectively) in Kolliphor^®^ EL/ethanol/normal saline (1:1:8 volume ratio) and adding Avastin^®^ to a final concentration of 1 mg/mL BVZ; Kolliphor^®^ EL was a kind gift from BASF (Ludwigshafen am Rhein, Germany). TMZ as powder was dissolved in the MIX formulation before use (0.32 mg/mL, 1.65 μM TMZ), to avoid pH-dependent degradation.

#### 4.1.2. Characterization

IL MIX droplets mean size, polydispersity index (PDI), and Zeta potential were determined 1 h after preparation using the dynamic light scattering technique (DLS; 90 Plus, Brookhaven, NY, USA). Size measurements were obtained at an angle of 90°, and Zeta potential at an angle of 15°, both at a temperature of 25 °C. All data were determined in triplicate. Droplet shape was determined through Transmission Electronic Microscopy (TEM, CH10, Philips, Amsterdam, The Netherlands) after negative staining with 1% phosphotungstic acid (Merck, Darmstadt, Germany) [37].

Drug recovery in IL MIX, defined as the ratio between the actual and the theoretical drug concentration, was assayed through RP-HPLC [46] after 2 step extraction. Briefly 50 μL IL MIX were diluted in 100 μL acetonitrile under vortex and centrifuged at 14,000 rpm (Allegra 64R centrifuge, Beckman Coulter, Brea, CA, USA), to extract TMZ-C12 and RAP. The precipitate was dissolved in 100 μL acetic acid, to extract the BVZ–AOT ion pair, and lipids were precipitated by adding 50 μL water, followed by 14,000 rpm centrifugation. BVZ–AOT extraction was performed prior to adding PS to the formulation, because the molecular size limit of RP-HPLC does not allow the detection of PS-BVZ-AOT complex. Drug recovery was assayed also in control formulations used for cell and animal experiments, owing to suitable RP-HPLC methods (see Supplementary Materials Section S4).

EE%, defined as the ratio between the drug within the lipid droplets and the total drug present in the formulation, was determined after size exclusion of IL MIX by employing Agarose CL 4B (Alfa-Aesar, Haverhill, MA, USA). EE% was calculated as the ratio between the drug recovery after and before size exclusion. RP-HPLC was used for TMZ-C12 and RAP quantification, and DS-PAGE electrophoresis coupled with densitometry for BVZ determination [47] (see Supplementary Materials Section S5-Figure S3 and Table S2).

Further evidence of BVZ entrapment in the lipid droplets was obtained by employing BVZ fluorescently labelled with FITC (Sigma-Aldrich, St. Louis, MO, USA) (BVZ-FITC), through FOM (DM2500, Leica Microsystems, Wetzlar, Germany) and asymmetrical FFF (AF2000, Postnova, Landsberg am Lech, Germany) [48,49]. The FFF methods employed are shown in the Supplementary Materials Section S6 (Figures S4.1 and S4.2, Table S3).

### 4.2. Cell Studies

PCF-2 and JR8 human melanoma cells were a kind gift of Dr. Pistoia (Gaslini Institute, Genoa, Italy). A2058 human melanoma cells and B16-F10 mouse melanoma cells were from the American Type Culture Collection (ATCC; Manassas, VA, USA).

Human Umbilical Vein Endothelial Cells (HUVEC) were isolated from human umbilical veins and maintained as previously described [50]. The use of HUVEC was approved by the Ethics Committee of the “Presidio Ospedaliero Martini” of Turin (263-07/NF) and conducted in accordance with the Declaration of Helsinki. Written informed consent was obtained from all donors.

#### 4.2.1. Internalization

The lipid matrix of IL was fluorescently labelled with 0.1 mg/mL 6-coumarin (Sigma-Aldrich, St. Louis, MO, USA), pre-dissolved in a small amount of DMSO and added to the nanoemulsion under vortex and ultrasound. Internalization of fluorescently labelled RAP (FITC–glicylrapamycin) and BVZ (BVZ–FITC) loaded in IL was studied by employing in house synthesized fluorescent drugs [51,52,53,54,55] (see Supplementary Materials Sections S7 and S8-Figure S6). BVZ–FITC final concentration in IL was 1 mg/mL, while FITC–glicylrapamycin final concentration in IL was 250 μg/mL. Melanoma cells were cultured as described below (Section 4.2.2), until a 10^6^ cells/mL concentration was reached. Then cells were incubated for 1 h with fluorescently labelled IL (1:100 dilution in cell medium) and IL loaded with FITC–glicylrapamycin and BVZ–FITC (1:10 dilution in cell medium). Different dilutions were employed owing to the fluorescence intensity of the formulations under study. Finally, cells were centrifuged and re-suspended in 0.5 mL PBS. Internalization was assessed through flow cytofluorimetry (C6 cytofluorimeter, Accuri Cytometers, Ann Arbor, MI, USA), and expressed as iMFI, that is the product of percent cell internalized and mean fluorescence intensity. Three different replicates were performed.

#### 4.2.2. Cytotoxicity: WST-1 Assay

A2058, PCF-2 and JR8 cell lines were cultured in Roswell Park Memorial Institute 1640 medium (RPMI 1640) and B16-F10 cell lines in Dulbecco’s Modified Eagle Medium (DMEM) (both from Sigma-Aldrich, St. Louis, MO, USA) with 10% Fetal Bovine Serum (FBS) (PAA Laboratories, Pasching, Austria), 2 mmol/L L-glutamine and penicillin/streptomycin (100 units/mL) (both from Sigma-Aldrich, St. Louis, MO, USA), at 37 °C in 5% CO_2_ humidified atmosphere. Cells (1 × 10^3^/well) were seeded in 96-well plates and incubated for 24 h. Then, they were treated with the formulations under study for 72 h. The cell proliferation reagent 2-(4-iodophenyl)-3-(4-nitrophenyl)-5-(2,4-disulfophenyl)-2H-tetrazolium, monosodium salt (WST-1) (Dojindo Molecular Technologies Inc., Kumamoto, Japan) was used, as described by the manufacturer’s protocol. Cells that had received no drug, as control, were normalized to 100%, and the readings from treated cells were expressed as percent of viability inhibition. Eight replicates were used to determine each data point and five different experiments were performed.

#### 4.2.3. Proliferation: Clonogenic Assay

Melanoma cells (8 × 10^2^/well) were seeded into six-well plates. The day after, they were treated with different concentrations of the studied drugs for 72 h. Then the medium was changed, and cells were cultured for additional 7 h in a drug-free medium. Subsequently, cells were fixed and stained with a solution of 80% crystal violet (Sigma-Aldrich, St. Louis, MO, USA) and 20% methanol. Colonies were then photographed. Then 30% *v*/*v* acetic acid was added to induce a complete dissolution of the crystal violet. Absorbance was recorded at 595 nm by a 96-well-plate ELISA reader. Five different experiments were performed.

#### 4.2.4. Migration: Boyden Chamber Assay

Since in the migration assay, drug concentrations that do not exert cytotoxic effects should be used, a preliminary cytotoxicity assay was performed as follows. 8 × 10^3^ HUVEC or B16-F10 were seeded in 96-well plates and treated at 37 °C, 5% CO_2_, for 6 h with the formulations under study. Subsequently, cells were fixed and stained with a solution of 80% crystal violet and 20% methanol. Then 30% *v*/*v* acetic acid was added to induce a complete dissolution of the crystal violet. Absorbance was recorded at 595 nm by a 96-well-plate ELISA reader. Five different experiments were performed. 

Then, in the Boyden chamber-migration assay, 8 × 10^3^ cells were plated onto the apical side of 50 μg/mL Matrigel (BD Biosciences, San Jose, CA, USA) coated filters (8.2 mm diameter and 0.5 μm pore size) in a serum-free medium with or without formulations under study. A medium containing FCS 20% or 10 ng/mL VEGF-α was placed in the baso-lateral chamber as a chemo-attractant. The chamber was incubated at 37 °C under 5% CO_2_. After 6 h, the cells on the apical side were wiped off with Q-tips. Cells on the bottom of the filter were stained with crystal-violet and counted (five fields of each triplicate filter) with an inverted microscope (magnification 100×). The results are expressed as the number of migrated cells. The control migration was 45 ± 4 cells per microscope field (number of replicates = 5).

#### 4.2.5. Angiogenesis: Tube Formation Assay

In the tube-formation assay, drug concentrations that do not exert cytotoxic effects were also used. To this aim, in preliminary experiments HUVEC were seeded in 96-well plates and treated at 37 °C, 5% CO_2_, for 6 h with the formulations under study. Crystal violet was used for cytotoxicity evaluation.

Then, HUVEC were seeded onto 48-well plates (5 × 10^4^/well) previously coated with 75 μL of growth factor-reduced Matrigel, in the absence or presence of the formulations under study. The morphology of the capillary-like structures formed by the HUVEC was analysed by an inverted microscope after 6 h of culture and photographed with a digital camera. Tube formation was analysed with an imaging system (Image Pro Plus Software for micro-imaging, Media Cybernetics, version 5.0, Bethesda, MD, USA). Tube formation was evaluated by counting the total number of tubes in three wells and five different experiments were performed, as previously described [56]. Cells that had received no drug, as control, were normalized to 100% of newly formed vessels and the readings from the treated cells were expressed as percent of vessel inhibition. Five different replicates were performed.

### 4.3. Animal Experiments

Female, 6- to 9-week-old C57BL6/J (The Jackson Laboratory, Bar Harbor, ME, USA) mice were bred under pathogen-free conditions in the animal facility of the University of Eastern Piedmont, Department of Health Sciences (Authorization n. 61/2005-A-6 May 2005, issued by the General Directorate of Veterinary and Food Health-Italian Ministry of Health), and treated in accordance with the University Ethical Committee and European guidelines (Experimental protocol authorization n. 477/2016-PR, released in 16-05-2016 from Italian Ministry of Health for protocol n. DB064.6 of 10-03-2016).

C57BL6/J mice were injected subcutaneously with B16-F10 cells (1 × 10^5^ in 100 μL/mouse) and the tumour growth was monitored every two days. Ten days after the tumour induction, the mice were treated with the i.v. injection of the formulation under study or the same volume of PBS as control. The treatment was carried out three times a week for two weeks (6 i.v./mouse) and the mice were sacrificed after three days after the last injection, or when they displayed sufferance. Employed doses were: TMZ 1.6 mg/kg, BVZ 5 mg/Kg, RAP low dose 1.25 μg/kg, RAP high dose 1.25 mg/kg. Tumour volume was monitored through the treatment period. Five animals for groups were employed for each group. At the end of the experiment, mice were sacrificed, and tumour mass and volume were measured. Experiments were performed with 6 mice per group.

#### 4.3.1. Immunohistochemistry on Tumour Specimens

Immediately after dissection, tumour samples were embedded in optimal cutting temperature (OCT) compound (Bio Optica Milano S.p.A., Milan, Italy) and stored at −80 °C until use. Tumour tissues were cut with a cryostat (thickness 4–5 µm) and treated with 4% paraformaldehyde diluted in PBS for 5 min at room temperature to fix the sample on the glass slides. The samples were then blocked with 5% normal goat serum (R&D Systems, Minneapolis, MN, USA) in PBS for 1 h to block aspecific sites to which the primary antibody could bind. To detect CD31 and Ki-67 expression, the primary antibodies used were a polyclonal rabbit anti-CD31 (Abcam, Cambridge, UK) or a monoclonal mouse anti-human Ki-67 antigen (Thermo Fisher Scientific, Waltham, MA, USA), both diluted 1:50 in PBS, and incubated over night at 4 °C in a humid chamber. Since Ki-67 is a nuclear antigen, it was necessary to permeabilize the samples by using PBS + 0.1% Triton X-100 (Sigma-Aldrich, St. Louis, MO, USA). The secondary antibody used was, respectively, an anti-rabbit Ig Alexa fluor 488-conjugated, or an anti-rat Ig Alexa fluor 546-conjugated (both from Thermo Fisher Scientific, Waltham, MA, USA), both diluted 1:400 in PBS. The sections then were stained with 0.5 mg/mL of the fluorescent dye 4,6-diamidino-2-phenylindole-dihydrochloride (DAPI) (Sigma-Aldrich, St. Louis, MO, USA) for 5 min to highlight cell nuclei and then mounted using prolong anti-fade mounting medium (SlowFade AntiFADE Kit, Molecular Probes Invitrogen, Carlsbad, CA, USA). The sections were then observed by a fluorescence microscope (DM2500, Leica, Italy) and analysed by Image Pro Plus Software for micro-imaging 5.0 (Media Cybernetics, version 5.0, Bethesda, MD, USA). Tumour microvessel density (TMD) was measured by evaluating the CD31-positive area, the numbers of positive cells for Ki-67, and the total tumour area per field upon slide scanning (Panoramic midi II, 3D Histech, Budapest, Hungary), as previously described [57,58]. The number of Ki-67 positive cells was evaluated in at least ten different fields, by manual counting.

#### 4.3.2. Real Time PCR on Tumours

Total RNA was isolated from tumours, using TRIzol reagent (Sigma-Aldrich, St. Louis, MO, USA). RNA (1 μg) was retrotranscribed using the QuantiTect Reverse Transcription Kit (Qiagen, Hilden, Germany). IFN-γ and IL-10 expression were evaluated with a gene expression assay (Assay-on-Demand; Applied Biosystems, Foster City, CA, USA). The GUSB gene was used to normalize the cDNA amounts. Real-time PCR was performed using the CFX96 System (Bio-Rad Laboratories, Hercules, CA, USA) in duplicate for each sample in a 10 μL final volume containing 1 μL of diluted cDNA, 5 μL of TaqMan Universal PCR Master Mix (Applied Biosystems, Foster City, CA, USA), and 0.5 μL of Assay-on-Demand mix. The results were analysed with a ΔΔ threshold cycle method.

### 4.4. Statistical Analysis

Data are shown as mean ± SEM. Statistical analyses were performed with Prism 3.0 software (GraphPad Software, La Jolla, CA, USA) using one-way ANOVA and the Dunnett’s test. Values of *p* ≤ 0.05 were considered statistically significant. Kaplan–Mayer survival curves were evaluated with the χ^2^ test.

## 5. Conclusions

In preliminary preclinical studies, nanoemulsions for total parenteral nutrition proved to be a biocompatible and versatile delivery system for advanced melanoma treatment, being suitable to load a combination of drugs of different chemical nature and pharmacological activity. The proposed poly-chemotherapy increases the efficacy against melanoma both in in vitro and in vivo models, likely by acting on multiple mechanisms.

## Figures and Tables

**Table 1:** IL MIX physico-chemical characterization. PDI: polydispersion Index; RP-HPLC: reverse phase high pressure liquid chromatography; SDS-PAGE: sodium dodecyl sulphate polyacrylamide gel electrophoresis; EE%: % entrapment efficiency.

**Mean Size (nm)**		262.6 ± 15
**PDI**		0.113
**Zeta Potential (mV)**		−33.14 ± 4.5
**Recovery**	RP-HPLC	TMZ 75 ± 4% RAP 100 ± 3% BVZ 64 ± 7%
	SDS-PAGE electrophoresis/densitometry	BVZ 57 ± 8%
**EE%**	RP-HPLC	TMZ 68 ± 4% RAP 56 ±6%
	SDS-PAGE electrophoresis/densitometry	BVZ 47 ± 5%

**Table 2:** iMFI calculated through flow cytometry. IL: Intralipid^®^; FITC: fluorescein isotiocyanate; BVZ-FITC: FITC labelled bevacizumab; iMFI: mean fluorescence intensity.

Formulations	iMFI (A2058 Cells)	iMFI (B16-F10 Cells)
Control	501.77	102.82
Fluorescently labelled IL	2,613,367.19	1,414,784.30
BVZ-FITC loaded IL	6,193.05	18,342.9
FITC-glycilrapamycin loaded IL	26,891.73	69,574.71

## Supplementary Materials

The following are available online at https://www.mdpi.com/2072-6694/12/5/1198/s1, Section S1: Stability of Nanoemulsion at Different pH; Section S2: Immunohistochemistry Images; Section S3: TMZ-C12 Synthesis; Section S4: RP-HPLC; Section S5: SDS-PAGE Electrophoresis; Section S6: FFF; Section S7: Fluorescent Labelling of BVZ: BVZ-FITC Preparation; Section S8: Fluorescent Labelling of RAP: FITC-Glicylrapamycin Synthesis; Figure S1.1: CD31 immunohistochemistry images (Magnification 200×); Figure S1.2: Ki67 immunohistochemistry images (Magnification 200×); Figure S2: Scheme of TMZ-C12 synthesis; Figure S3: Example of SDS PAGE electrophoresis of free BVZ and IL BVZ, before and after size exclusion and relative densitometry; Figure S4.1: FFF method and fractograms of free antibodies; Figure S4.2: FFF method and fractograms of BVZ-FITC loaded IL; Figure S5: SEC-HPLC chromatograms; Figure S6: Scheme of fluorescent RAP synthesis; Table S1: Mean diameter, PDI and Zeta potential of nanoemulsion at different pH; Table S2: Densitometry integration table of gel; Table S3: Calculated hydrodynamic radius of free antibodies (Zetasizer Nano); Table S4: Quantification of proteic components owing to SEC HPLC.
