# Supplementary material for: Nanoemulsions as Delivery Systems for Poly-Chemotherapy Aiming at Melanoma Treatment
Source: Cancers (Basel). 2020 May 9;12(5):1198. doi: 10.3390/cancers12051198 (PMC7281359; doi:10.3390/cancers12051198)
Supplement: Supplementary file 1 [file cancers-12-01198-s001.pdf]

Supplementary Materials

Nanoemulsions as Delivery Systems for Poly-Chemotherapy Aiming to Melanoma Treatment

Chiara Dianzani, Chiara Monge, Gianluca Miglio, Loredana Serpe, Katia Martina, Luigi Cangemi, Chiara Ferraris, Silvia Mioletti, Sara Osella, Casimiro Luca Gigliotti, Elena Boggio, Nausicaa Clemente, Umberto Dianzani and Luigi Battaglia

Section 1: Stability of Nanoemulsion at Different pH

Table S1. Mean diameter, PDI and Zeta potential of nanoemulsion at different pH.

Buffer pH	Mean Diameter (nm)	PDI	Zeta Potential (mV)
7.4	290.0	0.005	−48.05
6.0	270.0	0.128	−48.79
5.6	303.0	0.045	−40.19
5.0	284.4	0.118	−37.32

Section 2: Immunohistochemistry Images

(CTR)

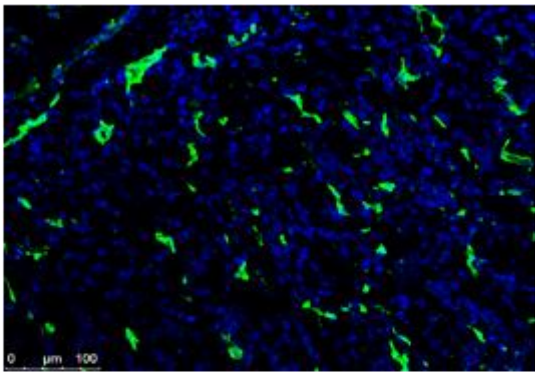

(A)

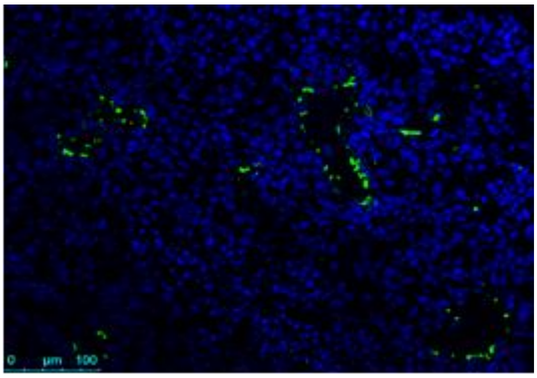

(B)

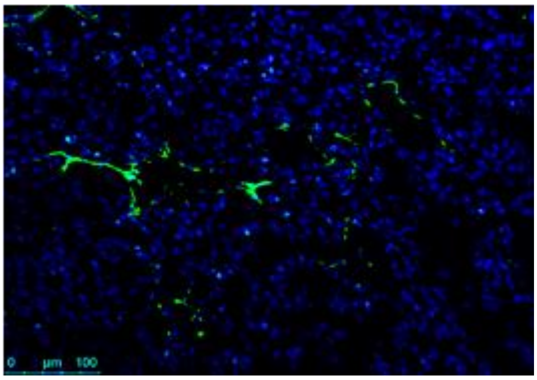

(C)

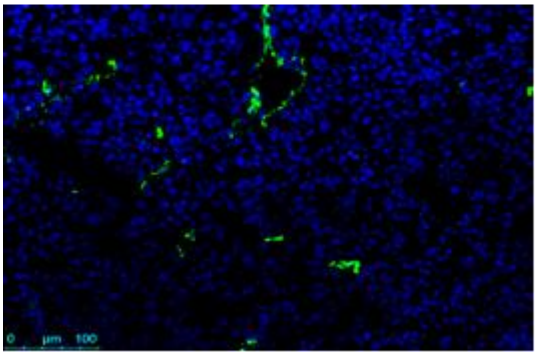

(D)

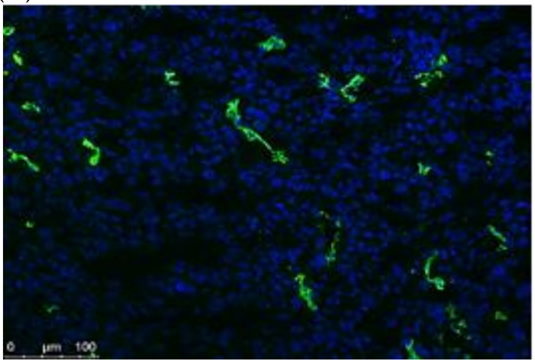

Figure S1.1. CD31 immunohistochemistry images (Magnification 200×). (**CTR**) control; (**A**) Intralipid® (IL) loaded with combination of drugs (MIX) and high dose of rapamycin (RAP); (**B**) IL MIX-RAP low dose; (**C**) MIX-RAP high dose; (**D**) MIX-RAP low dose.

(CTR)

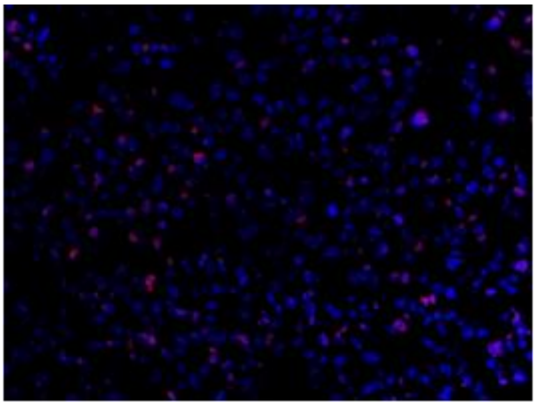

(A)

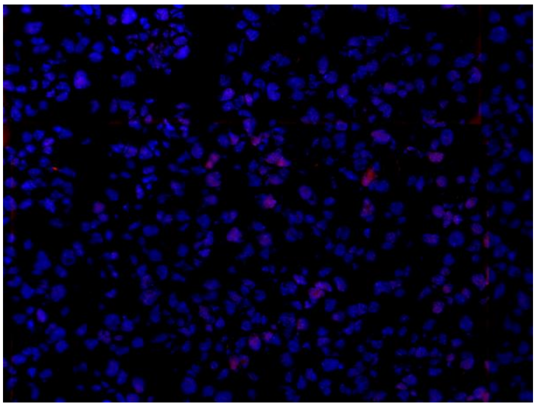

(B)

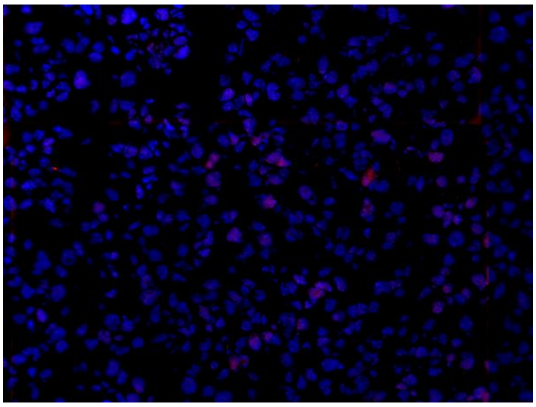

(C)

(D)

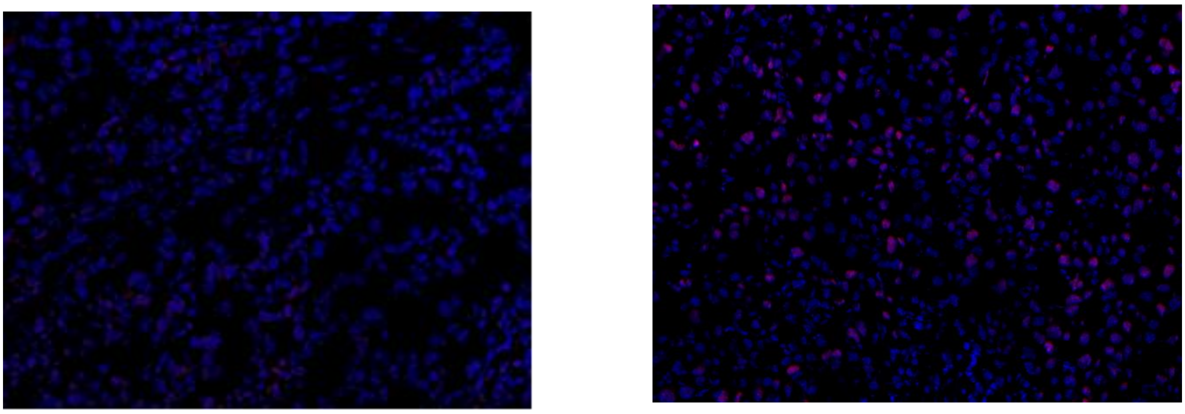

Figure S1.2. Ki67 immunohistochemistry images (Magnification 200×). (**CTR**) control; (**A**) Intralipid® (IL) loaded with combination of drugs (MIX) and high dose of rapamycin (RAP); (**B**) IL MIX-RAP low dose; (**C**) MIX-RAP high dose; (**D**) MIX-RAP low dose.

Section 3. TMZ-C12 Synthesis

The Synthesis of TMZ-C12 was performed in 2 steps according to literature methods [13,26,27] (Figure S2).

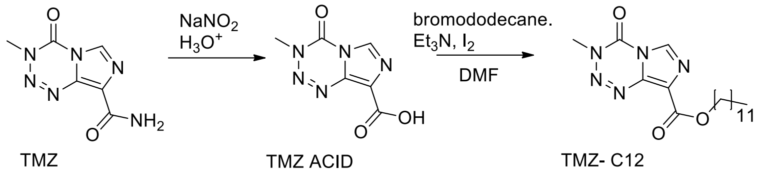

Figure S2. Scheme of TMZ-C12 synthesis.

Step 1: Synthesis of TMZ Acid

TMZ (0.1546 mmol, 30 mg) was solubilized in 0.24 mL of concentrated sulphuric acid, sodium nitrite (0.564 mmol, 39 mg) in 0.15 mL water was added dropwise in ice bath, and then the mixture was kept stirring overnight at room temperature. Ice water (2 mL) was added and the precipitated TMZ acid was collected and freeze-dried (23 mg).

Step 2: Synthesis of TMZ-C12

23 mg of TMZ acid (0.120 mmol, 23 mg), triethylamine (37 μL), bromododecane (0.240 mmol, 60 μL) (Carlo Erba, Val De Reuil, France) were dissolved in anhydrous DMF (246 μL) and a catalytic amount of iodine was added. The mixture was stirred for 4 h at 40 °C. After cooling down the reaction mixture, 750 μL of ethyl acetate were added and the organic solution washed with water (3 × 750 μL). The organic phase was dried under nitrogen steam, then the residue was dissolved in 1 mL dicholoromethane and purified by a silica gel flash column (0–50% ethyl acetate/dicholoromethane gradient) to give TMZ-C12, which was characterized by mass spectrometry (ESI-MS, TSQ 700, Finnigan-Mat) and HPLC (see below). ESI-MS $[M + H]^+$: m/z 364. Retention time in HPLC was 8.5 min. Total reaction yield was 30%.

Section 4: RP-HPLC

4.1. TMZ

Analyses were performed with a YL9100 HPLC system equipped with a YL9110 quaternary pump, a YL9101 vacuum degasser and a YL9160 PDA detector, linked to YL-Clarity software for data analysis (Young Lin, Hogyedong, Anyang, Korea). Column was a Teknocroma C18 mediterranea Sea 25 × 0.46 cm. Linear gradient (10 min) from 100% acetic acid to 50% acetonitrile, followed by 5 min at 50% acetonitrile was performed at a flow rate of 1 mL/min. PDA detector was set at 329 nm. Retention time was 9 min [13].

4.2. TMZ-C12

HPLC analysis was performed using a LC-6A pump, an SPD-6A UV-vis detector and a C-R6A integrator (Shimadzu, Tokyo, Japan). A reversed-phase column (Allsphere™ ODS, 2.5 μm 250 × 4.6 mm) was used. Acetonitrile/water (70/30 v/v) was used as a mobile phase with a flow rate of 1 mL/min. UV-vis detector was set at 329 nm. Retention time was 8.5 min [13].

4.3. RAP, Azidoacetylrapamycin, Glycilrapamicin

HPLC analysis was performed using a LC-6A pump, an SPD-6A UV-vis detector and a C-R6A integrator (Shimadzu, Tokyo, Japan). A Beckmann ODS 25 × 0.5 cm column was employed. Methanol/water (75/25 v/v) was used as a mobile phase with a flow rate of 1 mL/min and at 57 °C temperature. UV-Vis detector was set at 277 nm. Retention times were 10.9 min for RAP, 19.5 min for azidoacetylrapamycin, and 8.4 min for glycilrapamycin.

4.4. FITC-Glicylrapamycin

A YL9100 HPLC system equipped with a YL9110 quaternary pump, a YL9101 vacuum degasser and a YL9160 PDA detector, linked to YL-Clarity software for data analysis (Young Lin, Hogyedong, Anyang, Korea) were used. Column was a Beckmann ODS 25 × 0.5 cm. A methanol/water gradient was performed with a flow rate of 1 mL/min and at 57 °C temperature: 25% to 75% methanol in 10 min, 75% methanol for 15 min, 75% to 25% methanol in 2 min. PDA wavelengths were 277 and 490 nm. Retention time was 13.1 min.

4.5. TMZ-C12, RAP, BVZ

The analysis was performed by modifying a literature method [29]. A YL9100 HPLC system equipped with a YL9110 quaternary pump, a YL9101 vacuum degasser and a YL9160 PDA detector, linked to YL-Clarity software for data analysis (Young Lin, Hogyedong, Anyang, Korea) were used. Column was a 300 nm pore size C8 Tecnokroma Tracer Excel 25 × 0.4 cm. A gradient was performed at 75 °C and 1 mL/min flow rate between eluent A (0.1% trifluoroacetic acid (TFA)) and eluent B (79% isopropanol, 20% acetonitrile, 10% water, 0.1% TFA): 0 min–90% A; 15 min–40% A; 20 min–40% A; 21 min–90%A. PDA wavelengths were 220 nm (BVZ), 277 nm (RAP), and 329 nm (TMZ-C12). Retention times were 10.9 min for BVZ, 16.0 min for RAP and 18.0 min for TMZ-C12.

Section 5: SDS-PAGE Electrophoresis

A sample with 5μL of ColorBurst™ Electrophoresis Marker (Sigma-Aldrich, St. Louis, MO, USA) and 5 μL of each sample were electrophoresed at 180 V on 12% acrylamide gel for 60 min using a Tris–glycine running buffer. Separated proteins were stained with the silver staining protocol of Mortz and co-workers (Figure S3) [30].

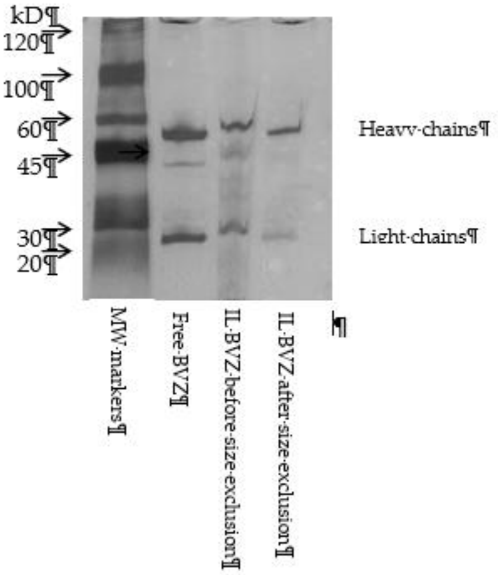

Figure S3. Example of SDS PAGE electrophoresis of free BVZ and IL BVZ, before and after size exclusion and relative densitometry.

Table S2. Densitometry integration table of gel.

	Free BVZ (Standard)	IL BVZ Before Size Exclusion	IL BVZ After Size Exclusion
50 KDa band	21.937.229	4.196.255	5.596.953
25 KDa band	16.277.430	2.451.790	5.325.761
total	38.214.659	6.648.045	10.922.714

Section 6: FFF

Asymmetrical FFF measurements were performed with an AF2000 FFF coupled with a UV-vis detector (Postnova, Landsberg am Lech, Germany), a Zetasizer Nano (Malvern Instruments, Malvern, UK), and a RF-551 fluorimeter (Shimadzu, Kyoto, Japan). Mobile phase was PBS. UV-vis detector was set at 280 nm, while fluorimeter λ_{exc} was 490 nm and λ_{em} was 520 nm. Samples were injected through a 20 µL loop: a concentration of 5 mg/mL was employed to compare free antibodies; pure IL (1 mg/mL loaded BVZ-FITC) was compared with 1 mg/mL free BVZ-FITC.

Free BVZ and BVZ-FITC analysis (Zetasizer Nano and UV-vis detectors)

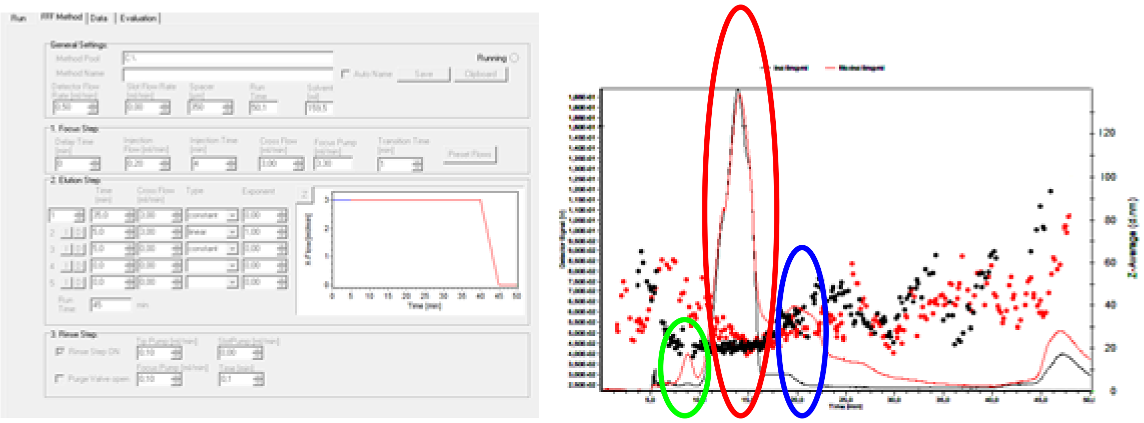

(A) Elution method

(B) Fractograms

Black signal: BVZ. Red: BVZ-FITC
Line: UV-vis signal intensity
Dots: mean size (Zetasizer)
Red circle: antibody monomer
Blue circle: antibody dimer and aggregates
Green circle: antibody fragments

Figure S4.1. FFF method and fractograms of free antibodies. (**A**) Elution method; (**B**) Fractograms.

Table S3. Calculated hydrodynamic radius of free antibodies (Zetasizer Nano).

Antibody	Size (nm)			
	aggregates	dimer	monomer	fragments
BVZ		35.5	21.4	47.7
BVZ-FITC	40.4	33.3	24.6	38.6

Antibody dimer and aggregates elute later compared to monomer, according to literature fractogram [31]. Hydrodynamic radius increases along with antibody aggregation state (Table S3). As previously reported in literature [32], detected hydrodynamic radius of fragments is larger compared to plain antibody, probably because of the different hydration state (Figure S4.1).

BVZ-FIC loaded IL vs. free BVZ-FITC (Zetasizer Nano, Fluorimeter and UV-Vis detectors)

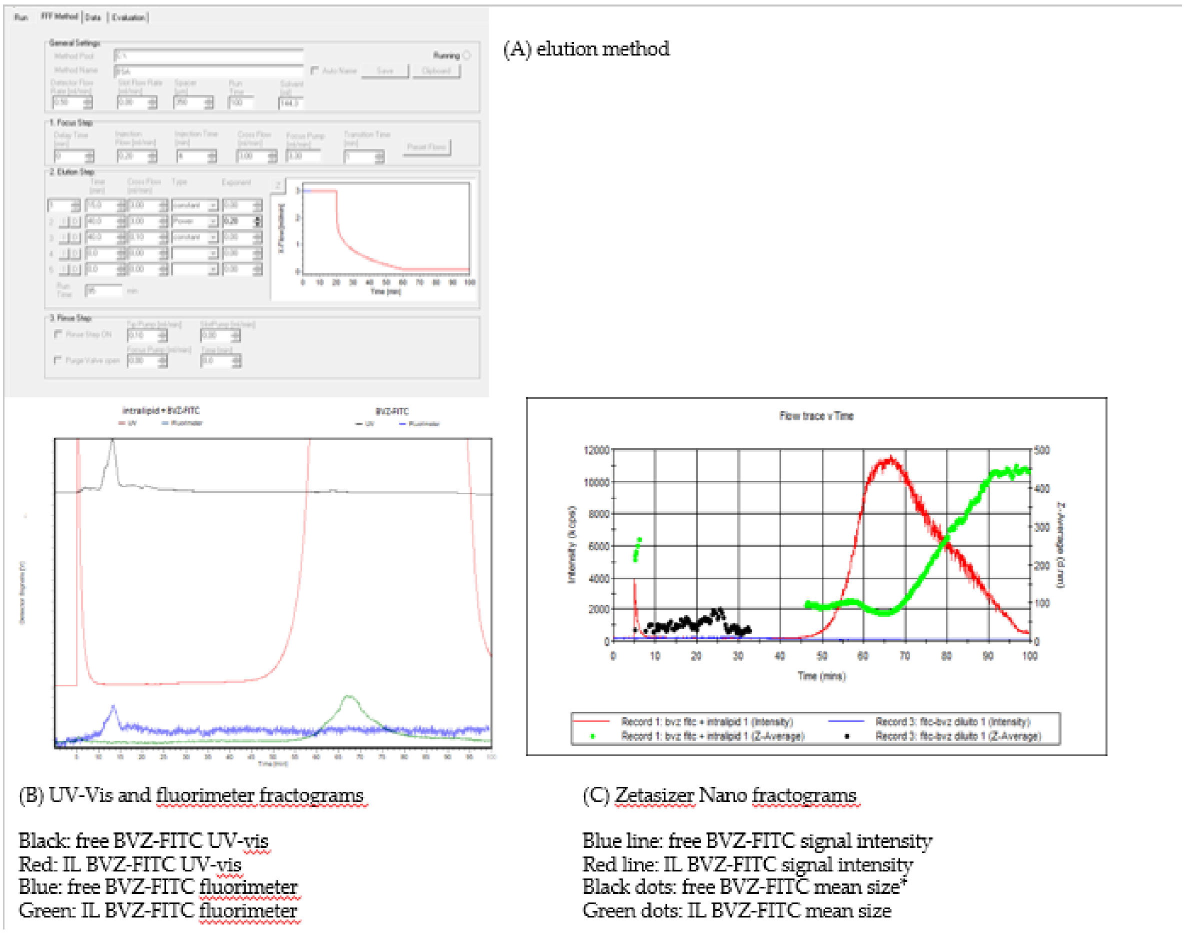

Figure S4.2. FFF method and fractograms of BVZ-FITC loaded IL. * By employing this elution method monomer and dimer of the antibody cannot be separed and only the signal of the aggregates (40.4 nm mean size) can be detected. (**A**) elution method; (**B**) UV-Vis and fluorimeter fractograms; (**C**) Zetasizer Nano fractograms.

In IL BVZ-FITC fractogram (Figure S1.1), fluorescence signal from BVZ-FITC meets UV-vis/Light Scattering signal from IL nanodroplets, thus confirming the effective loading of the antibody in IL

Section 7: Fluorescent Labelling of BVZ: BVZ-FITC Preparation

BVZ was derivatized with FITC to obtain a fluorescently labelled molecule [18,34]. Briefly, 250 µL of a 1 mg/mL FITC solution in 0.1 M pH = 10.5 carbonate buffer was added to 1 mL of a 25 mg/mL BVZ solution in a pH = 10.5 buffer. The reaction mixture was stored under stirring at room temperature for 2 h, protected from light by aluminium foil. BVZ-FITC was then purified from free FITC that had not reacted by eluting the reaction mixture through a Sephadex® G25 column (Sigma-Aldrich, St. Louis, MO, USA).

Starting BVZ and BVZ-FITC were characterized by FFF (see below) and SEC HPLC in order to investigate their integrity and aggregation state. SEC HPLC chromatograph consisted of a pump (LC 10-AD), a UV-Vis detector (Jasco UV-1575) and a data station (Cromatoplus, Aqualis, Milan, Italy). Column was a TSKgel 3000SWXL (Tosoh Bioscience, Rivoli, Italy). Mobile phase was 0.1 M sodium sulfate/0.1 M phosphate buffer pH 6.7. Flow rate was 1 mL/min. UV-vis detector was set at 220 nm. Samples were injected through a 20 µL loop at a concentration of 0.5 mg/mL. Obtained chromatograms are shown below (Figure S5 and Table S4).

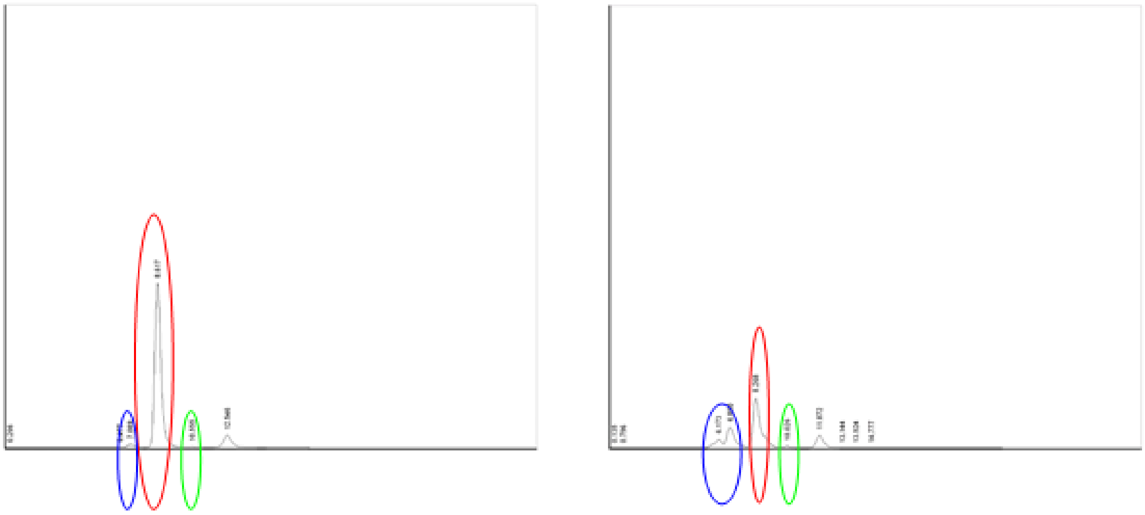

(A) BVZ (B) BVZ-FITC

Figure S5. SEC-HPLC chromatograms. Red circle: BVZ monomer. Blue circle: BVZ dimer and aggregates. Green circle: fragments. (**A**) BVZ; (**B**) BVZ-FITC

Table S4. Quantification of proteic components owing to SEC HPLC.

Antibody	Aggregates	Dimer	Monomer	Fragments
BVZ (Avastin®)	-	2.4%	97%	0.2%
BVZ-FITC	11%	26%	61%	1.7%

As expected, aggregation increases in labelled antibody, compared to parent molecule, probably owing to the labelling procedure and buffer exchange.

Section 8: Fluorescent Labelling of RAP: FITC-Glicylrapamycin Synthesis

Fluorescent labelling of RAP was performed through a multi-step reaction (Figure S6).

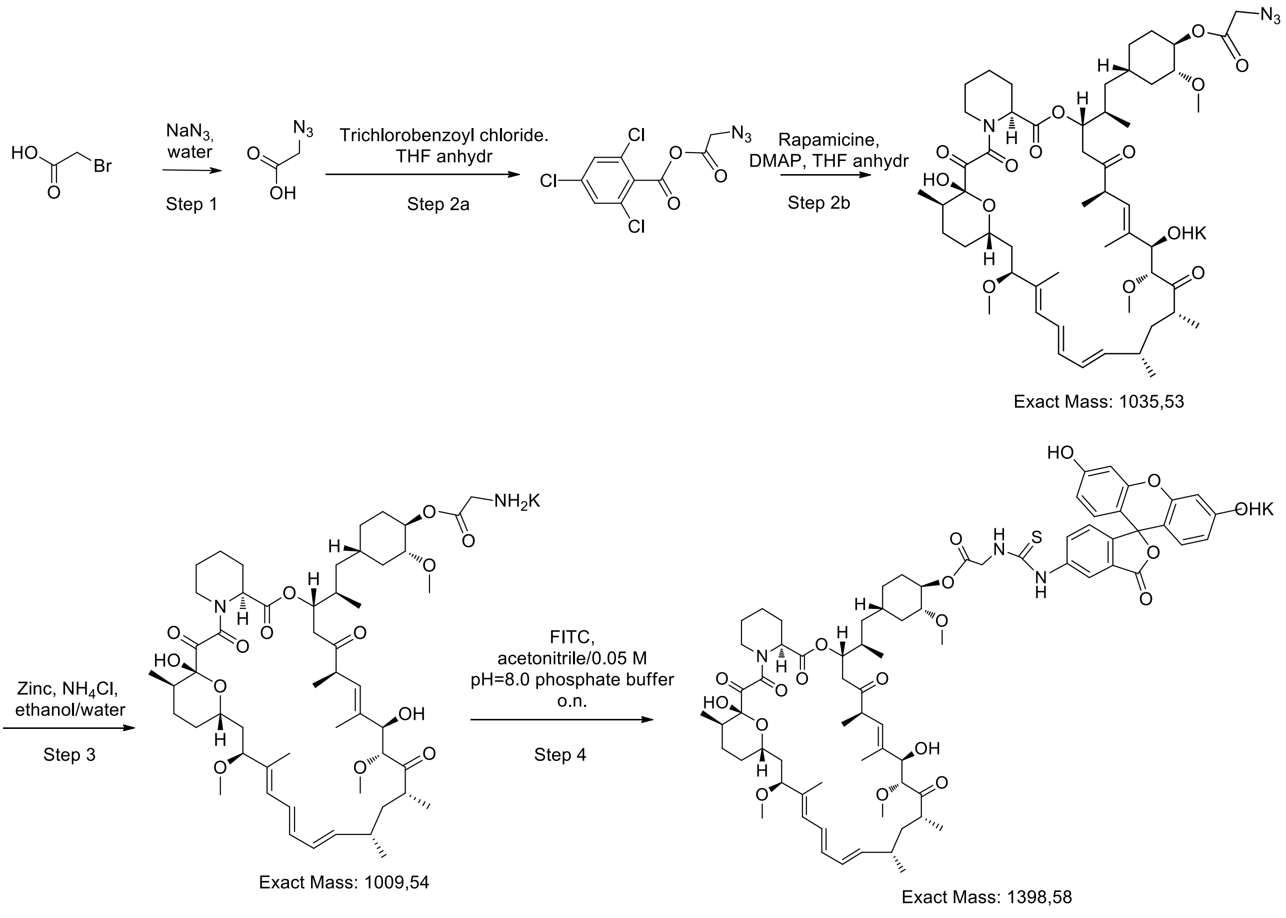

Figure S6. Scheme of fluorescent RAP synthesis.

Step 1: Synthesis of Azidoacetic acid

3.51 g of sodium azide (54 mmole, 10 equiv) (Sigma-Aldrich, St. Louis, MO, USA), 750 mg of bromoacetic acid (5.4 mmoles, 1 equiv) (Sigma-Aldrich, St. Louis, MO, USA) and 3.465 g of ammonium chloride (64.77 mmoles, 12 equiv) were dissolved in 15 mL water and kept reacting overnight at 60 °C. Reaction mixture was brought to pH = 4.0 with hydrochloric acid, and azidoacetic acid was extracted with diethyl ether (5 × 10 mL), then the organic phase was dried under nitrogen steam, obtaining a yellowish liquid with 1.350 g/mL density at 25 °C [35].

Step 2a and Step 2b: Synthesis of Azidoacetilrapamycin

Yamaguchi reaction for activating the azidoacetic acid to RAP coupling was performed [36]. Azidoacetic acid (5.4 µL, 7.3 mg, 72 µmoles, 1 equiv) was dissolved in 350 µL anhydrous tetrahydrofuran (THF) and brought to −20 °C. Then 11.3 µL (72 µmoles, 1 equiv) of trichlorobenzoyl chloride (TCB; Sigma-Aldrich, St. Louis, MO, USA) and 13.9 µL (98.8 µmoles, 1 equiv) of triethylamine were added. Reaction mixture was kept at −20 °C for 90 min, to obtain an anhydride as an activated form of azidoacetic acid. Reaction mixture becomes turbid upon formation of insoluble triethylammonium chloride [37]. The reaction mixture was brought at room temperature, then 60 mg of RAP (65 µmoles, 0.9 equiv) and 8.8 mg of 4-dimethylaminopyridine (72.5 µmoles, 1 equiv) (DMAP; Sigma-Aldrich, St. Louis, MO, USA) were added. Azidoacetic anhydride reacts with RAP forming azidoacetylrapamycin [37]. The reaction was stopped by addition of 1 mL of sodium carbonate 0.1 M and extracted with chloroform (3 × 1 mL). Organic phase was dried with anhydrous sodium sulphate and dried under nitrogen steam. Then the residue was dissolved in 1 mL chloroform and purified by a silica gel flash column (0–3% methanol/choloroform gradient).

Step 3: Synthesis of Glycilrapamicin

Reduction of azidoacetylrapamicin was performed with zinc and ammonium chloride [38]. Briefly, a stock solution was prepared by dissolving 80 mg ammonium chloride in 1.6 mL ethanol and 0.54 mL water. Then 20.5 mg (20.5 μmoles, 1.36 equiv) azidoacetylrapamycin were reduced with an excess of 12.3 mg of zinc (188.6 μmoles, 12.57 equiv), suspended in 102.5 μL of the aforementioned stock solution (20 min at 60 °C). Un-reacted azidoacetylrapamycin was separated by diluting the reaction mixture with 2 parts of 0.1 M hydrochloric acid and centrifuging at 26,000 rpm (Allegra 64R centrifuge, Beckman Coulter, Brea, CA, USA), and it underwent further reduction steps until depletion. Glycilrapamycin was extracted with chloroform from the clear supernatant, which was previously diluted with 1 part of 0.1 M sodium carbonate. Organic phase was dried under nitrogen steam.

Step 4: Labelling of Glicylrapamycin with FITC

10 mg (10.3 μmoles, 1.67 equiv) glicylrapamycin and 4 mg (10.3 μmoles, 1.67 equiv) FITC were dissolved in 1 mL acetonitrile and 0.5 mL 0.05 M pH = 8.0 phosphate buffer and kept reacting overnight. Then reaction mixture was diluted with 2.17 mL 0.1 M hydrochloric acid, in order to precipitate FITC-glycilrapamycin, which was dried under vacuum.

RAP, azidoacetilrapamycin, glicylrapamycin and FITC-glycilrapamycin were characterized through HPLC (see sections 4.3 and 4.4) and ESI-MS.

ESI-MS spectra were recorded on a Micromass Quattro API micro (Waters Corporation, Milford, MA, USA) mass spectrometer. Data were processed using a MassLynx System (Waters).

Azidoacetylrapamycin: MW = 996.57 m/z calc $[M + H]^+$ = 997.57 found $[M + H]^+$ = 997.7; $[M + Na]^+$ =1019.6 and $[M + K]^+$ =1035.62.

Glicylrapamycin: MW = 970.58; m/z calc $[M + H]^+$ = 971.58 found $[M + H]^+$ m/z = 971.8 $[M + Na]^+$ m/z = 993.7 and $[M + K]^+$ m/z = 1009.6.

FITC-glicylrapamycin: MW = 1359.61; m/z calc $[M + H]^+$ = 1360.61 found $[M + H]^+$ = 1360.8 $[M + Na]^+$ = 1382.7 and $[M + 39]^+$ = 1398.7.

Total reaction yield was 6.5%.

© 2019 by the authors. Licensee MDPI, Basel, Switzerland. This article is an open access article distributed under the terms and conditions of the Creative Commons Attribution (CC BY) license (http://creativecommons.org/licenses/by/4.0/).
